# The Japanese Early-Stage Trial of High-Dose Methylcobalamin for Amyotrophic Lateral Sclerosis (JETALS): Protocol for a Randomized Controlled Trial

**DOI:** 10.2196/12046

**Published:** 2018-12-21

**Authors:** Ryosuke Oki, Yuishin Izumi, Hiroyuki Nodera, Yasutaka Sato, Hiroshi Nokihara, Kazuaki Kanai, Masahiro Sonoo, Makoto Urushitani, Kazuto Nishinaka, Naoki Atsuta, Nobuo Kohara, Toshio Shimizu, Hitoshi Kikuchi, Masaya Oda, Ken Ikeda, Makiko Nagai, Kiyonobu Komai, Yasuhiro Kojima, Daisuke Kuzume, Sagiri Isose, Shun Shimohama, Koji Abe, Hidefumi Ito, Kazuyuki Noda, Tomohiko Ishihara, Mitsuya Morita, Takayoshi Shimohata, Satoshi Teramukai, Tatsuo Kagimura, Kensuke Noma, Hiroaki Yanagawa, Satoshi Kuwabara, Ryuji Kaji, JETALS

**Affiliations:** 1 Department of Clinical Neuroscience Institute of Biomedical Sciences Tokushima University Graduate School Tokushima Japan; 2 Clinical Trial Center for Developmental Therapeutics Tokushima University Hospital Tokushima Japan; 3 Department of Neurology Juntendo University School of Medicine Tokyo Japan; 4 Department of Neurology Teikyo University School of Medicine Tokyo Japan; 5 Department of Neurology Shiga University of Medical Science Otsu Japan; 6 Department of Neurology Sumitomo Hospital Osaka Japan; 7 Department of Neurology Nagoya University Graduate School of Medicine Nagoya Japan; 8 Department of Neurology Kobe City Medical Centre General Hospital Kobe Japan; 9 Department of Neurology Tokyo Metropolitan Neurological Hospital Tokyo Japan; 10 Department of Neurology Murakami Karindo Hospital Fukuoka Japan; 11 Department of Neurology Vihara Hananosato Hospital Miyoshi Japan; 12 Department of Neurology Toho University Omori Medical Center Tokyo Japan; 13 Department of Neurology Kitasato University East Hospital Sagamihara Japan; 14 Department of Neurology National Hospital Organization Iou Hospital Kanazawa Japan; 15 Department of Neurology Takeda General Hospital Kyoto Japan; 16 Department of Neurology Chikamori Hospital Kochi Japan; 17 Department of Neurology National Hospital Organization Chiba-East Hospital Chiba Japan; 18 Department of Neurology Sapporo Medical University Sapporo Japan; 19 Department of Neurology Graduate School of Medicine, Dentistry and Pharmaceutical Sciences Okayama University Okayama Japan; 20 Department of Neurology Wakayama Medical University Wakayama Japan; 21 Department of Neurology Juntendo University Shizuoka Hospital Izunokuni Japan; 22 Department of Neurology Brain Research Institute Niigata University Niigata Japan; 23 Division of Neurology Department of Internal Medicine Jichi Medical University Tochigi Japan; 24 Department of Neurology and Geriatrics Gifu University Graduate School of Medicine Gifu Japan; 25 Department of Biostatistics Graduate School of Medical Science Kyoto Prefectural University of Medicine Kyoto Japan; 26 Translational Research Center for Medical Innovation Foundation for Biomedical Research and Innovation Kobe Japan; 27 Division of Regeneration and Medicine Medical Center for Translational and Clinical Research Hiroshima University Hospital Hiroshima Japan; 28 Department of Cardiovascular Regeneration and Medicine Research Institute for Radiation Biology and Medicine Hiroshima University Hiroshima Japan; 29 Department of Neurology Graduate School of Medicine Chiba University Chiba Japan

**Keywords:** amyotrophic lateral sclerosis, methylcobalamin, vitamin B12, JETALS, clinical trial

## Abstract

**Background:**

Amyotrophic lateral sclerosis (ALS) is a neurodegenerative disease that affects the upper and lower motor neurons. Currently, only riluzole and edaravone are approved as drugs to treat ALS and new agents with larger effect sizes are warranted. Exploratory analyses in our previous study (study ID #E0302-J081-761) have suggested that high-dose methylcobalamin (E0302) prolonged the overall survival of ALS patients and suppressed ALS progression in patients with a disease duration of less than 12 months.

**Objective:**

This clinical trial aims to evaluate the efficacy and safety of E0302 for treatment of ALS patients within one year of onset.

**Methods:**

The Japanese early-stage trial of high-dose methylcobalamin for ALS (JETALS) is a prospective, multicenter, placebo-controlled, double-blind, randomized phase III study conducted at 24 tertiary neurology centers and is funded by the Japan Agency for Medical Research and Development. A total of 128 ALS patients within one year of onset were randomized at a 1:1 ratio to receive intramuscular injection with E0302 50 mg or placebo twice a week for 16 weeks. The primary endpoint is changes in the ALS Functional Rating Scale-Revised (ALSFRS-R) total score at 16 weeks. If patients wish to receive E0302 50 mg after the double-blind administration period, E0302 will be provided to them until March 2020 during the continuous administration period.

**Results:**

This study began in October 2017 and is being conducted at 24 participating institutions in Japan. The study is in progress and the patient enrollment period is scheduled to end in August 2019, with follow-up scheduled to end in March 2020.

**Conclusions:**

This study is being performed to revalidate the efficacy and safety of E0302 in patients with early-stage ALS in the first year of symptom onset. If positive results are obtained, the aim is to apply for E0302 approval as a new drug for the treatment of ALS.

**Trial Registration:**

ClinicalTrials.gov NCT03548311; https://clinicaltrials.gov/ct2/show/NCT03548311 (Archived by WebCite at http://www.webcitation.org/74Fw3rDzb)

**International Registered Report Identifier (IRRID):**

PRR1-10.2196/12046

## Introduction

Amyotrophic lateral sclerosis (ALS) is a disease of unknown etiology that affects upper and lower motor neurons and results in progressive systemic muscle weakness and atrophy. The median survival time—period from onset until the use of an invasive respiratory support device or death—is 20-48 months [1].

Many drugs have been evaluated in clinical trials for treating patients with ALS; however, apart from riluzole and edaravone, none have been approved by the US Food and Drug Administration. Riluzole has been shown to prolong survival by 2-3 months [2]. Edaravone has been shown to slow the advance of the ALS Functional Rating Scale-Revised (ALSFRS-R) score: the least-squares mean difference during 24 weeks between the edaravone group and the placebo group was 2.49 (SE 0.76, 95% CI 0.99-3.98; *P*=.001) in favor of edaravone [3].

Hence, the development of a drug that extends the survival time or ameliorates clinical symptoms of ALS is widely anticipated. High-dose methylcobalamin—an active form of vitamin B12—has been suggested to have a protective effect against neurodegeneration in vitro and in vivo [4-7]. Clinical research reports have suggested that administration of high-dose methylcobalamin to patients with ALS has yielded clinically beneficial results [8,9].

In one study, methylcobalamin (0.5 or 25 mg/day) was intramuscularly administered for 14 days to 24 patients with ALS [8]. As a result, the compound muscle action potential amplitude in the 25 mg methylcobalamin group was significantly increased after 4 weeks—2 weeks after administration was completed—compared with that before administration of methylcobalamin.

The aforementioned nonclinical and clinical research results showed that high-dose methylcobalamin may be an effective treatment option for ALS. Owing to the favorable safety and pharmacokinetic results in the phase I clinical study, the subsequent phase II and III clinical studies targeting Japanese patients with ALS (study ID #E0302-J081-761) were conducted by Eisai Co, Ltd, hereinafter referred to as Eisai [9]. In that study, patients who were diagnosed with definite, probable, or probable-laboratory-supported ALS using revised El Escorial criteria within three years after symptom onset were enrolled and randomly assigned to receive placebo, E0302 (methylcobalamin) 25 mg, or E0302 50 mg intramuscularly twice weekly for 182 weeks. Primary endpoints were event-free survival (ie, time until death, invasive respiratory support device, or all-day noninvasive respiratory support device) and ALSFRS-R changes. In their study, 370 patients were analyzed—placebo (n=123), E0302 25 mg (n=124), and E0302 50 mg (n=123). Results showed that the E0302 25 mg and 50 mg groups had a tendency to surpass the placebo group with respect to ALSFRS-R total score as one of the primary endpoints, but did not go as far as indicating statistical significance.

In another study, 144 patients who were given a diagnosis of ALS within 12 months after symptom onset were analyzed—placebo (n=48), E0302 25 mg (n=54), and E0302 50 mg (n=42) [10]. The median event-free survival was 570 days, 1087 days, and 1197 days, respectively, which was prolonged in a dose-dependent manner (E0302 25 mg hazard ratio 0.64, 95% CI 0.38-1.09; E0302 50 mg hazard ratio 0.50, 95% CI 0.27-0.93; *P*=.01). In addition, ALSFRS-R changes were smaller in both E0302 groups (E0302 25 mg plus E0302 50 mg, *P*=.003; E0302 50 mg, *P*=.01) [10]. Although this observation was noted only in the results of the subpopulation analysis, the fact that high-dose methylcobalamin showed high efficacy in patients with ALS who were diagnosed early and registered in the study is considered clinically significant.

In this study, the incidence of adverse events was high, but the incidence of adverse reactions was limited. No obvious difference was observed between the placebo, the E0302 25 mg, and the E0302 50 mg groups in the occurrence of adverse events or reactions and there was no issue with the safety of intramuscular administration of E0302 25 mg or E0302 50 mg. One case of death by cardiac arrest was observed as a serious adverse reaction in the E0302 50 mg group, which was determined to be “possibly related” to the investigational product, since this association cannot be completely ruled out.

Based on the results of the phase II and III clinical studies conducted by Eisai, we planned to conduct an investigator-initiated trial to confirm the efficacy and safety of E0302 50 mg for treatment of ALS patients within one year after onset. In this trial, we adopted the updated Awaji criteria for the first time in the world, to our knowledge, which has displayed higher sensitivity compared to the El Escorial/revised Airlie House diagnostic criteria [11]. We adopted the updated Awaji criteria because we needed to increase the registration rate as much as possible, due to the enrollment of the early-stage patients. Our primary endpoint was the variation in the ALSFRS-R total score from allocation day to 16 weeks of treatment.

## Methods

### Trial Design Overview

**Figure 1:**
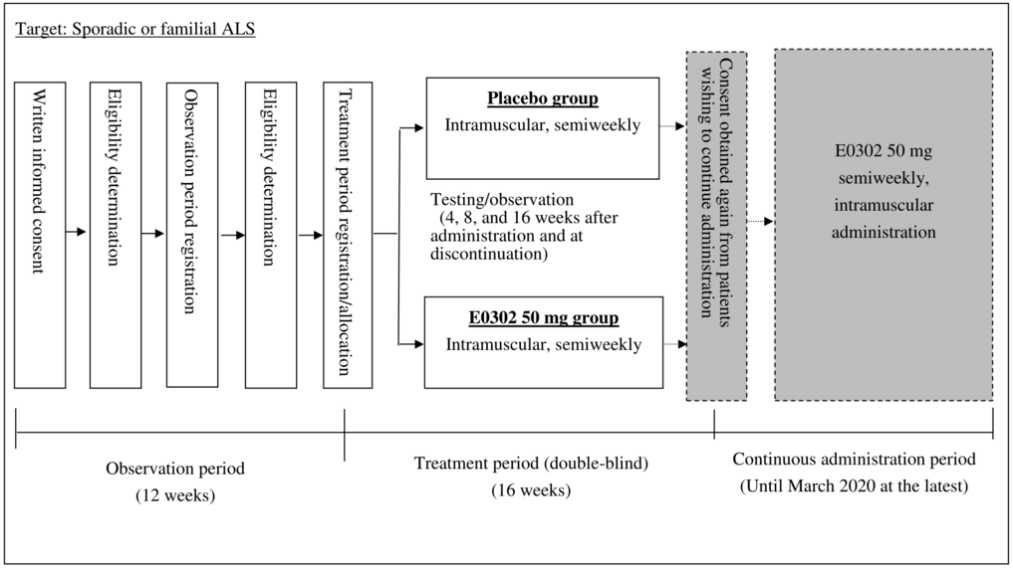
Schematic depiction of the trial design. The study is composed of three periods: the observation period, treatment period, and continuous administration period. ALS: amyotrophic lateral sclerosis; E0302: methylcobalamin.

This Japanese early-stage trial of high-dose methylcobalamin for ALS (JETALS) is a multicenter, randomized, placebo-controlled, double-blind, parallel-group comparative study. It comprises three study periods: observation, treatment, and continuing administration (see Figure 1). The eligibility of participants who registered for the observation period will be determined at the end of the 12-week observation period. Those confirmed as eligible will be registered in the treatment period and allocated to the placebo group or the E0302 50 mg group through dynamic allocation.

Randomization will be performed using a central registration system. Allocation will be done using a modification of the minimization method, which is a dynamic allocation method used to avoid selection bias in medical institutions. The investigational product will be allocated according to the patient registration sequence, taking into consideration the following characteristics: disease type (ie, bulbar onset, upper-limb onset, and lower-limb onset); ALS severity at the end of the observation period (ie, grade 1 or 2 according to Japan ALS severity classification, which ranges from grade 1 to 5, with grade 5 being the most severe); time from symptom onset to the start of the observation period (ie, ≤9 months or >9 months and ≤12 months); forced vital capacity (FVC) at the end of the observation period (ie, <90% or ≥ 90%), and edaravone administration history (ie, no or yes). These patient characteristics will be used as the allocation adjustment factors (ie, minimization factors) in consideration of the balance of groups within each facility and the overall balance.

E0302 50 mg or placebo will be intramuscularly administered twice weekly from the administration start date until the end of the 16-week treatment period. Investigational product administration and its efficacy, safety, and ALSFRS-R assessment will be conducted by various independent personnel to ensure as much blinding as possible during the treatment period. The efficacy, ALSFRS-R, and safety assessors will conduct necessary assessments at 4, 8, and 16 weeks after the start of investigational product administration, as well as at the time of event onset and at the time of discontinuation. All ALSFRS-R assessors underwent an ALSFRS-R training program, following the procedure manual made for this study.

The primary endpoint is a variation in the ALSFRS-R total score from allocation day until week 16 of treatment. Safety endpoints are adverse events, laboratory test results, electrocardiogram measurements, and vital sign measurements.

Subjects who wish to continue administration after week 16 of treatment will be transferred to the continuing administration period and they may continue treatment with E0302 50 mg until March 2020 at the latest. Independent assessment will not be necessary during the continuing administration period as investigators will conduct safety and efficacy assessments. The patients’ self-administration and the administration by their families and home-visit nurses at their homes will also be allowed in the continuing administration period.

### Eligibility Criteria

Target patients are those who were diagnosed with sporadic or familial ALS corresponding to the categories of definite, probable, or probable-laboratory-supported in the updated Awaji criteria (see Table 1) [11]. Inclusion and exclusion criteria are listed in Textbox 1; patients who satisfy all of the criteria will be included.

Patients will be considered as eligible participants according to the following: those who satisfied inclusion criteria 1-4, 6, and 7 when the observation period started; those who satisfied inclusion criteria 3 and 5-7 when the observation period was completed; those who did not meet any of the exclusion criteria when the observation period started; and those who did not meet exclusion criteria 1-4, 7-11, 13, and 14 when the observation period was completed.

### Interventions

During the treatment period, the patients will be administered with E0302 50 mg/day, or a placebo intramuscularly twice weekly. During the continuous administration period, patients will be administered with E0302 50 mg/day, depending on each patient’s request.

### Outcomes

The primary endpoint is a variation in the ALSFRS-R total score from allocation day until week 16 of the treatment period. The secondary endpoints are the time from allocation day until the onset of an event (ie, 24-hour use of noninvasive respiratory support equipment, use of invasive respiratory support, or death) as well as variation in FVC, blood homocysteine concentration, the manual muscle test total score, grip strength (both left and right), the Norris scale total score, and the Amyotrophic Lateral Sclerosis Assessment Questionnaire-40 (ALSAQ-40) total score. The safety endpoints are adverse events, laboratory test results, electrocardiogram measurements, and vital sign measurements.

The endpoints during the continuing administration period are the time from allocation day until the onset of an adverse event (ie, 24-hour use of noninvasive respiratory support equipment, use of invasive respiratory support, or death) as well as variation in the ALSFRS-R total score, laboratory test results, electrocardiogram measurements, vital sign measurements, and adverse events.

### Sample Size and Trial Duration

The target number of subjects is 128—placebo group (n=64) and E0302 50 mg group (n=64). In the previous study (ie, study ID #E0302-J081-761), the difference in the ALSFRS-R total score was estimated using data from a subpopulation of patients who were within one year of symptom onset when the observation period started and whose ALSFRS-R total score decreased by 1-2 points during the observation period (ie, 12 weeks) [10]. Based on the results of this subgroup analysis, we hypothesized that the E0302 50 mg group’s score would exceed that of the placebo group by the difference in the ALSFRS-R total scores. The required number of subjects to set the type I error probability to ≤2.5% in the one-sided tests and to set the statistical power to ≥80% was a minimum of 60 patients per group, based on the subgroup population result. Considering that there will be discontinuations during the study, the target number of patients for this study was determined to be 64 patients per group.

The planned patient registration period is from October 2017 to September 2019; the planned study implementation period is from October 2017 to March 2020.

**Table 1:** Updated Awaji criteria.

Diagnosis grade	Criteria
Definite	Clinical or neurophysiological evidence of upper and lower motor neuron dysfunction in the bulbar region and in at least two spinal regions or three spinal regions
Probable	Clinical or neurophysiological evidence of upper and lower motor neuron dysfunction in at least two regions, with some upper motor neuron signs necessarily rostral (above) to lower motor neuron dysfunction
Probable-laboratory-supported	Clinical signs of upper and lower motor neuron dysfunction in one region together with neurophysiological evidence of lower motor neuron dysfunction in two regions
Possible	Clinical or neurophysiological evidence of upper and lower motor neuron dysfunction in one region or upper motor neuron signs evident in two regions or lower motor neuron dysfunction evident rostral (above) to upper motor neuron signs

**Inclusion and exclusion criteria for the study.**

**Inclusion criteria:**

1. Patients from whom written consent to participate in this study was received

2. Patients who are ≥20 years of age at the time of providing informed consent

3. Patients diagnosed with sporadic or familial amyotrophic lateral sclerosis (ALS) corresponding to the categories of definite, probable, or probable-supported in the updated Awaji criteria

4. Patients who are within one year of symptom onset when the observation period started

5. Patients whose ALSFRS-R (Amyotrophic Lateral Sclerosis Functional Rating Scale-Revised) total score has decreased by 12 points during the observation period (12 weeks)

6. Patients who are rated as 1 or 2 according to Japan ALS severity classification (grade 1-5, grade 5 most severe)

7. Patients seen on an outpatient basis

**Exclusion criteria:**

1. Patients who have undergone a tracheostomy

2. Patients who are using a noninvasive respiratory support device

3. Patients with ≤60% forced vital capacity

4. Patients with chronic obstructive pulmonary disorder

5. Patients with vitamin B12 deficiency-based neurological symptoms

6. Patients who have received edaravone within four weeks prior to observation period registration

7. Patients who have started riluzole, changed the dosage, or discontinued the medication after giving informed consent

8. Patients with cognitive impairment

9. Patients who are or may be pregnant

10. Patients with a serious respiratory disorder, cardiovascular disease, or liver or kidney disease

11. Patients with a malignant tumor

12. Patients who have participated in another trial within the 12 weeks prior to giving informed consent

13. Patients with current illness or those with a history of drug allergy or severe allergic disease (anaphylactic shock)

14. Patients who are determined to be unsuitable for this study by the investigator or subinvestigator

### Statistical Considerations

The analysis sets for efficacy analysis are *full analysis set* and *per protocol set*. Full analysis set includes subjects who received the investigational product at least one time; this is the primary analysis set. Per protocol set is the secondary analysis set for confirming consistency of the results obtained from full analysis set from a sensitivity analysis standpoint. The *safety analysis set* refers to the set of subjects registered for the treatment period, excluding those who have no assessable safety data.

For the analysis of the primary endpoint, the difference in the ALSFRS-R scores from allocation day to each time point is the response variable; the administration groups, time points, minimization factors, and interaction between the administration groups and time points are the fixed effects. The ALSFRS-R total score on allocation day is the covariate and fits the linear model—Mixed-Model Repeated Measure analysis—with an unstructured covariance structure of the error variance. If the lower limit of the 95% confidence interval of the least mean square is over zero for the difference in the variation in the ALSFRS-R total scores at week 16 when comparing the placebo and E0302 50 mg groups, then it will be determined to be statistically significant. The significance level of the efficacy endpoints is set at one-tailed 2.5%.

### Ethics and Dissemination

The study protocol was approved by the Institutional Review Board at each site before the start of the trial. Substantial amendments of the study protocol must be approved by each Institutional Review Board. The trial was registered at ClinicalTrials.gov (NCT03548311) and the University Hospital Medical Information Network clinical trials registry (UMIN000029588). Written informed consent will be obtained directly from each patient.

## Results

This study began in October 2017 and is being conducted at 24 participating institutions in Japan. The study is in progress and the patient enrollment period is scheduled to end in August 2019, with follow-up scheduled to end in March 2020.

## Discussion

JETALS is a study that aims to verify the superiority of high-dose E0302 (methylcobalamin, 50 mg) intramuscular administration over placebo and to examine its safety in patients with ALS using the Japanese version of the ALSFRS-R as an indicator. Two revisions were made to the protocol of a previous study (study ID #E0302-J081-761), which was conducted as a preliminary study, targeting patients with ALS who were within one year of symptom onset.

The first revision changed the primary endpoint to a variation in the ALSFRS-R total score from allocation day to week 16 of treatment. The ALSFRS-R is a clinical assessment scale created to objectively and quantitatively assess the progress of patients with ALS. This scale can be used to clinically assess the motor function of the extremities, bulbar function, and respiratory function disorders. The ALSFRS-R score is frequently used as the primary endpoint in recent clinical studies targeting ALS. Because the ALSFRS-R is highly reliable for assessing ALS clinical symptoms using both the total score and item scores, and it can be used in clinical assessments, a variation in the ALSFRS-R total score was selected as the primary endpoint of this study [12]. Variations between different assessors may be minimized by having each assessor master the scale in advance through a training program. In our previous study (study ID #E0302-J081-761), the ALSFRS-R scores were evaluated at 4 and 16 weeks. For the patients who were given a diagnosis of ALS within 12 months after symptom onset, we predicted that after 16 weeks of treatment, it would be possible to detect a significant difference (data not shown). In addition, the ratio of ALSFRS-R scores between first symptom and first examination, during whole disease or within 100 days, correlates with survival time [13], hence the ALSFRS-R score at 16 weeks is thought to be a clinically important predictive factor of survival time. That is why we set the period of primary outcome evaluation at 16 weeks, from the points of view of effectiveness and ethics.

The second modification was changing the diagnostic criteria from the El Escorial/revised Airlie House diagnostic criteria to the updated Awaji criteria. The El Escorial/revised Airlie House diagnostic criteria are widely accepted, but their low diagnostic sensitivity has been considered an issue [14]. The reason for this low sensitivity can be attributed to the few cases in which widespread denervation is triggered in the early phase. In addition, another disadvantage of the El Escorial/revised Airlie House diagnostic criteria is that fasciculation potential, which is observed in the early phase of ALS, is not included in the electrodiagnostic criteria. The updated Awaji diagnostic criteria are advocated as an algorithm for combining the Awaji electrodiagnostic criteria and the El Escorial/revised Airlie House diagnostic criteria without variance [11]. In other words, the same framework as the El Escorial/revised Airlie House diagnostic criteria is maintained (ie, definite, probable, probable-laboratory-supported, and possible) and fasciculation potential is included as evidence of active denervation.

Geevasinga et al examined the testing sensitivity of each set of diagnostic criteria for 881 patients with ALS from 8 articles reported previously [11]. Data from 7 out of these 8 (88%) articles indicated that applying the updated Awaji diagnostic criteria over the El Escorial/revised Airlie House diagnostic criteria increased sensitivity, implying that the updated Awaji diagnostic criteria are definitely more advantageous.

This study targeted patients with ALS who were within one year of onset. Therefore, we used the updated Awaji diagnostic criteria, with its increased diagnostic sensitivity. Effects on efficacy assessment when using diagnostic criteria that differs from that used in the previous study needed to be confirmed. To this end, an analysis was conducted that narrowed down subpopulations corresponding to clinically definite, probable, and probable-laboratory-supported ALS based on the El Escorial/revised Airlie House diagnostic criteria. This was done when the observation period was completed and the effect of the criteria on the primary endpoint was examined.

Currently, only riluzole and edaravone have been approved as treatment drugs for ALS worldwide, but their effects are limited. As with the results of the subpopulation analysis, E0302 prolonged event-free survival more than 600 days and slowed the advance of the ALSFRS-R total score by 3.3 during 16 weeks; its safety and tolerability was well-established in the previous study [10]. Therefore, E0302 is highly anticipated as a new drug for treating ALS. E0302 can be used not only for monotherapy, but for multitherapy with riluzole and edaravone, which could change the strategy of ALS treatment.

## Abbreviations

ALS: amyotrophic lateral sclerosis
ALSAQ-40: Amyotrophic Lateral Sclerosis Assessment Questionnaire-40
ALSFRS-R: Amyotrophic Lateral Sclerosis Functional Rating Scale-Revised
E0302: methylcobalamin
FVC: forced vital capacity
JETALS: Japanese early-stage trial of high-dose methylcobalamin for ALS
